# Inequalities between degree- and distance-based graph invariants

**DOI:** 10.1186/s13660-018-1633-y

**Published:** 2018-02-15

**Authors:** Imran Nadeem, Hani Shaker

**Affiliations:** COMSATS Institute of Information Technology, Lahore, Pakistan

**Keywords:** 05C90, 92E10, Inequalities, Topological indices, Degree-based index, Distance-based index

## Abstract

Inequalities provide a way to study topological indices relatively. There are two major classes of topological indices: degree-based and distance-based indices. In this paper we provide a relative study of these classes and derive inequalities between degree-based indices such as Randić connectivity, *GA*, *ABC*, and harmonic indices and distance-based indices such as eccentric connectivity, connective eccentric, augmented eccentric connectivity, Wiener, and third *ABC* indices.

## Introduction

Let $G=(V_{G},E_{G})$ be a simple connected graph with a vertex set $V_{G}$ and an edge set $E_{G}$. The numbers of vertices and edges of *G* are respectively called the order *n* and the size *m* of *G*. The number of edges incident to a vertex $p\in V_{G}$ is called the degree of *p* and is denoted by $d_{p}$. $M_{p}$ represents the product of degrees of all vertices of *G* which are adjacent to the vertex *p*, i.e., $M_{p}=\prod_{pq\in E_{G}}d_{q}$. The minimum and maximum degrees of graph *G* are respectively denoted by *δ* and Δ. If all the vertices of *G* are of the same degree *d*, then *G* is termed a regular graph of degree *d*. The distance from a vertex $p\in V_{G}$ to a vertex $q\in V_{G}$ is denoted by $d(p,q)$ and is defined as the minimum number of edges lying between them. The eccentricity of a given vertex $p\in V_{G}$ is denoted by $\varepsilon_{p}$ and is defined as the maximum distance between *p* and any other vertex $q\in V_{G}$. The maximum and minimum eccentricities of *G* are called the diameter $d_{G}$ and the radius $r_{G}$ of *G*. If all vertices of *G* are of the same eccentricity, then *G* is termed a self-centered graph and otherwise a non-self-centered graph.

A topological index is a numerical quantity which is uniquely determined for a graph and invariant under graph isomorphism. Topological indices are extensively used in chemistry as molecular descriptors. This molecular descriptor provides a convenient and efficient way of translating the chemical constitution of a molecule into a numerical value by using the graph representation of the molecule called the molecular graph. This graph invariant can be used for correlation with the physical properties of that molecule. Several topological indices are extensively used to study the quantitative structure–activity (QSAR) and structure–property (QSPR) relationships [1–3].

Generally, topological indices can be categorized in two major classes: one is degree-based indices and the other is distance-based indices. The general expression for the class of degree-based indices can be defined as

1 $$ \mathit{DTI}(G)=\sum_{pq\in E_{G}} F(d_{p},d_{q}). $$

Milan Randić [4] presented the first degree-based topological index, called the Randić connectivity index $(\chi)$, which is defined from (1) by taking $F(d_{p},d_{q})=1/\sqrt{d_{p}\cdot d_{q}}$. Estrada et al. [5] presented the atom-bond connectivity index (*ABC*) which is formulated by setting $F(d_{p},d_{q})=\sqrt{(d_{p}+d_{q}-2)/d_{p}\cdot d_{q}}$ in (1). Vukicevic et al. [6] presented another degree-based topological index, called the geometric-arithmetic (*GA*) index, which is defined from (1) by choosing the function $F(d_{p},d_{q})=2\sqrt{d_{p}\cdot d_{q}}/(d_{p}+d_{q})$. The harmonic index (*H*) is presented in [7] and is defined from (1) by taking $F(d_{p},d_{q})=2/(d_{p}+d_{q})$.

The first distance-based index was introduced by the chemist Harold Wiener [8] and is called the Wiener index, defined as

$$ W(G)=\sum_{\{p,q\}\subseteq V_{G}}d(p,q). $$

The eccentricity related topological indices belong to the class of distance-based indices. The general expressions for these indices can be defined in the following ways:

2 $$\begin{aligned}& \mathit{ETI}_{1}(G)=\sum_{p\in V_{G}} F(d_{p},\varepsilon_{p}), \end{aligned}$$

3 $$\begin{aligned}& \mathit{ETI}_{2}(G)=\sum_{p\in V_{G}} F(M_{p},\varepsilon_{p}), \end{aligned}$$

4 $$\begin{aligned}& \mathit{ETI}_{3}(G)=\sum_{pq\in E_{G}} F(\varepsilon_{p},\varepsilon_{q}). \end{aligned}$$

The first eccentricity related topological index presented by Sharma et al. [9] is called the eccentric connectivity index $(\xi^{c})$ and is defined from (2) by taking $F(d_{p},\varepsilon_{p})=d_{p}\cdot \varepsilon_{p}$. Gupta et al. [10] presented an eccentricity related index called the connective eccentric index ($C^{\xi}$), which is also formulated from (2) by setting $F(d_{p},\varepsilon_{p})=d_{p}/\varepsilon_{p}$. Another eccentricity related index, called the augmented eccentric connectivity index ($\xi^{ac}$), was presented by Dureja et al. [11] and is defined by choosing $F(M_{p},\varepsilon_{p})=M_{p}/\varepsilon_{p}$ in (3). Dae-Won Lee [12] presented the third atom-bond connectivity index $(\mathit{ABC}_{3})$ which is defined from (4) by taking the function $F(\varepsilon_{p},\varepsilon_{q})=\sqrt{(\varepsilon_{p}+\varepsilon _{q}-2)/\varepsilon_{p}\cdot \varepsilon_{q}}$.

Inequalities provide a way to study topological indices relatively. This relative study is being conducted in three directions. One direction is to study inequalities for a topological index; this includes upper/lower bounds of topological index and inequalities for topological index between a graph and its associated transformed graph. Ji et al. [13] characterized the upper and lower bounds for the reformulated Zagreb index for trees, unicyclic and bicyclic graphs. Gao et al. [14] derived the sharp upper and lower bounds for the hyper-Zagreb index for trees, unicyclic and bicyclic graphs. In [13, 14], they used the inequality relations between a graph and its associated transformed graphs for the said indices. Wang et al. [15] presented the inequalities for general sum-connectivity indices between a graph and its several graph transformations.

The second direction is to study inequality relations between two different topological indices which belong to the same class. Lokesha et al. [16] derived some inequality relations between Randić and *GA* indices. Ali et al. [17] studied the inequality relations between various degree-based indices. From the class of distance-based indices, Dankelmann et al. [18] presented the inequality relation between Wiener and eccentric connectivity indices. Das et al. [19] derived the inequality relation between eccentric connectivity and Szeged indices.

The third and most significant direction is to study inequalities between topological indices which belong to two different classes. Hua et al. [20] derived the inequality relations of eccentric connectivity with the Zagreb indices. Zhou et al. [21] presented the inequalities between Wiener, hyper-Wiener, and Zagreb indices. Das et al. [22] derived the inequality relations between certain degree and distance-based topological indices.

In this paper we emphasize the relative study of topological indices belonging to two different classes. In this paper, we establish inequality relations of some degree-based indices such as Randić, *GA*, *ABC*, and harmonic indices with various distance-based indices such as eccentric connectivity, connective eccentric, augmented eccentric connectivity, Wiener, and third *ABC* indices.

## Preliminaries

In this section, we recall some preliminary results for the topological indices of a connected graph related to some graph parameters, i.e., the order, the size, the radius, and the diameter.

The relation between the diameter and the radius of a connected graph is presented in the following theorem.

### Theorem 1

([23])

*Consider a connected graph having radius* $r_{G}$ *and diameter* $d_{G}$, *then*

5 $$ r_{G}\leq d_{G}\leq2r_{G} $$

*and the left equality holds iff* *G* *is a self*-*centered graph*.

The lower and upper bounds of the eccentric connectivity index related to the radius and the diameter, respectively, are given in the following theorem.

### Theorem 2

([24])

*Consider a connected graph* *G* *having size* *m*, *radius* $r_{G}$, *and diameter* $d_{G}$, *then*

6 $$ 2mr_{G}\leq\xi^{c}(G)\leq2md_{G}, $$

*and the equality holds iff* *G* *is a self*-*centered graph*.

The lower and upper bounds of the connective eccentric index related to the diameter and the radius, respectively, are given in the following result.

### Theorem 3

([25])

*Consider a connected graph* *G* *having size* *m*, *radius* $r_{G}$, *and diameter* $d_{G}$, *then*

7 $$ \frac{2m}{d_{G}}\leq C^{\xi}(G)\leq\frac{2m}{r_{G}}, $$

*and the equality holds iff* *G* *is a self*-*centered graph*.

The lower and upper bounds of the augmented eccentric connectivity index related to the diameter and the radius, respectively, are presented in the following result.

### Theorem 4

([26])

*Consider a connected graph* *G* *having order* *n*, *radius* $r_{G}$, *diameter* $d_{G}$, *minimum degree* *δ*, *and maximum degree* Δ, *then*

8 $$ \frac{\delta^{\delta}}{d_{G}}\leq\frac{1}{n}\cdot\xi^{ac}(G) \leq \frac{\Delta^{\Delta}}{r_{G}}, $$

*and the equality holds iff* *G* *is a regular self*-*centered graph*.

### Theorem 5

([12])

*Consider a connected graph* *G* *having size* *m*, *radius* $r_{G}\geq2$, *and diameter* $d_{G}$, *then*

9 $$ \frac{\sqrt{2}m}{d_{G}}\sqrt{d_{G}-1}\leq \mathit{ABC}_{3}(G)\leq \frac{\sqrt{2}m}{r_{G}}\sqrt{r_{G}-1}, $$

*and the equality holds iff* *G* *is a self*-*centered graph*.

The inequality between the eccentric connectivity index and the Wiener index is presented in the following result.

### Theorem 6

([18])

*Consider a connected graph* *G* *having order* $n\geq3$, *then*

10 $$ W(G)\leq\frac{2}{3}n\xi^{c}(G)-n+1. $$

The relation between the Randić connectivity index and the diameter is given in the following result.

### Theorem 7

([27])

*Consider a connected graph* *G* *having order* $n\geq3$, *then*

11 $$ R(G)-\frac{1}{2}d_{G}\geq\sqrt{2}-1, $$

*and the equality holds iff* $G \cong P_{n}$.

The relation between the *ABC* index and the radius is given in the following result.

### Theorem 8

([28])

*Consider a connected graph* *G* *having order* $n\geq2$, *then*

12 $$ \mathit{ABC}(G)- r_{G}\geq \frac{n-1}{\sqrt{2}}- \biggl\lfloor \frac{n}{2} \biggr\rfloor , $$

*and the equality holds iff* $G \cong P_{n}$ *for* $n\geq3$.

The relation between the harmonic index and the diameter is given in the coming result.

### Theorem 9

([29])

*Consider a connected graph* *G* *having order* $n\geq4$ *and diameter* $d_{G}$, *then*

13 $$ H(G)-d_{G}\leq\frac{n}{2}-1, $$

*and the equality holds iff* $G\simeq K_{n}$.

The relations of *GA* index with Randić connectivity and harmonic indices are given in the following results.

### Theorem 10

([17])

*Consider a connected graph* *G* *having order* $n\geq3$, *then*

14 $$ \sqrt{\frac{4}{3}}R(G)\leq \mathit{GA}(G) \leq(n-1)R(G), $$

*the left and right equalities hold iff* $G\cong P_{3}$ *and* $G\cong K_{n}$, *respectively*.

### Theorem 11

([17])

*Consider a connected graph* *G* *having order* $n\geq2$, *then*

15 $$ H(G)\leq \mathit{GA}(G) \leq(n-1)H(G), $$

*the left and right equalities hold iff* $G\cong P_{n}$ *and* $G\cong K_{n}$, *respectively*.

## Main results

In this section, we establish the inequality relations between the class of some degree-based indices with the class of certain distance-based indices.

### Randić and *GA* indices in relation with distance-based indices

In the following theorem, we derive inequalities between the Randić connectivity index and certain distance-based indices such as eccentric connectivity, connective eccentric, augmented eccentric connectivity, and Wiener indices.

#### Theorem 12

*Consider a connected graph* *G* *having order* $n\geq3$ *and size* *m*, *then*

$$\begin{aligned} (\mathrm{a})&\quad R(G)> \frac{\xi^{c}(G)}{4m}+\sqrt{2}-1, \\ (\mathrm{b})&\quad R(G)> \frac{m}{C^{\xi}(G)}+\sqrt{2}-1, \\ (\mathrm{c})&\quad R(G)> \frac{n\cdot \delta^{\delta}}{2\xi^{ac}(G)}+\sqrt{2}-1, \\ (\mathrm{d})&\quad 8mnR(G)> 3W(G)+8mn(\sqrt{2}-1)+3(n-1), \end{aligned}$$

*where* *δ* *denotes the minimum degree of* *G*.

#### Proof

By considering the functions $F(d_{p},\varepsilon_{p})=d_{p}\cdot\varepsilon_{p}$, $F(d_{p},\varepsilon_{p})=d_{p}/\varepsilon_{p}$ and $F(M_{p},\varepsilon_{p})=M_{p}/\varepsilon_{p}$ in the general expressions for eccentricity related topological indices of any connected graph *G* as given in (2) and (3), we have eccentric connectivity, connective eccentric, and augmented eccentric connectivity indices of *G*, respectively, as follows:

$$\begin{aligned}& \xi^{c}(G) = \sum_{p\in V_{G}}d_{p} \cdot\varepsilon_{p}, \\& C^{\xi}(G) = \sum_{p\in V_{G}}d_{p}/ \varepsilon_{p}, \\& \xi^{ac}(G) = \sum_{p\in V_{G}}M_{p}/ \varepsilon_{p}. \end{aligned}$$

From (6)–(8), we have the relations of these indices with the diameter of *G* as follows:

16 $$\begin{aligned}& \frac{\xi^{c}(G)}{2m}\leq d_{G}, \end{aligned}$$

17 $$\begin{aligned}& \frac{2m}{C^{\xi}(G)}\leq d_{G}, \end{aligned}$$

and the equality holds in each of inequalities (16)–(17) iff *G* is a self-centered graph. Also,

18 $$ \frac{n\cdot\delta^{\delta}}{\xi^{ac}(G)}\leq d_{G}, $$

and the equality holds iff *G* is a regular self-centered graph.

Now, by considering the function $F(d_{p},d_{q})=1/\sqrt{d_{p}\cdot d_{q}}$ in the general expression of degree-based indices as given in (1), we have the Randić connectivity index of *G* as follows:

$$ R(G)=\sum_{pq\in E_{G}}\frac{1}{\sqrt{d_{p}\cdot d_{q}}}. $$

From (11), we have the relation of this index with the diameter of *G* as follows:

19 $$ d_{G}\leq2R(G)+2-2\sqrt{2}, $$

and the equality holds iff $G\cong P_{n}$ for $n\geq3$.

By combining inequality (19) with inequalities (16)–(18), we have

$$\begin{aligned}& \frac{\xi^{c}(G)}{2m}< 2R(G)+2-2\sqrt{2}, \\& \frac{2m}{C^{\xi}(G)}< 2R(G)+2-2\sqrt{2}, \\& \frac{n\cdot \delta^{\delta}}{\xi^{ac}(G)}< 2R(G)+2-2\sqrt{2}. \end{aligned}$$

The equality does not hold in each of these inequalities because if $G\cong P_{n}$ for $n\geq3$, then *G* cannot be a self-centered graph. After simplification, we get the required results (a), (b), and (c).

Also, from (10) we have the relation between Wiener and eccentric connectivity indices as follows:

20 $$ \xi^{c}(G)\geq\frac{3}{2n} \bigl(W(G)+n-1 \bigr); $$

and from result (a), we obtain

$$ 4mR(G)+4m-4\sqrt{2}m> \xi^{c}(G). $$

By combining this inequality with inequality (20), we get the required result (d). □

In the following corollary, we establish inequalities between the *GA* index and certain distance-based indices such as eccentric connectivity, connective eccentric, augmented eccentric connectivity, and Wiener indices.

#### Corollary 1

*Consider a connected graph* *G* *having order* $n\geq3$ *and size* *m*, *then*

$$\begin{aligned} (\mathrm{a})&\quad \mathit{GA}(G)> \frac{1}{\sqrt{12}}\frac{\xi^{c}(G)}{m}+2\sqrt{ \frac {2}{3}}-\frac{2}{\sqrt{3}}, \\ (\mathrm{b})&\quad \mathit{GA}(G)> \frac{2}{\sqrt{3}}\frac{m}{C^{\xi}(G)}+2\sqrt{ \frac {2}{3}}-\frac{2}{\sqrt{3}}, \\ (\mathrm{c})&\quad \mathit{GA}(G)> \frac{1}{\sqrt{3}}\frac{n\cdot \delta^{\delta}}{\xi ^{ac}(G)}+2\sqrt{ \frac{2}{3}}-\frac{2}{\sqrt{3}}, \\ (\mathrm{d})&\quad 4mn\mathit{GA}(G)> \sqrt{3}W(G)+\frac{8}{\sqrt{3}}mn( \sqrt{2}-1)+\sqrt{3}(n-1), \end{aligned}$$

*where* *δ* *denotes the minimum degree of* *G*.

#### Proof

By considering the function $F(d_{p},d_{q})=2\sqrt{d_{p}\cdot d_{q}}/(d_{p}+d_{q})$ in the general expression of degree-based indices given in (1), we have the *GA* index of *G* as follows:

$$ \mathit{GA}(G)=\sum_{pq\in E_{G}}\frac{2\sqrt{d_{p}\cdot d_{q}}}{d_{p}+d_{q}}. $$

From the compound inequality (14), we have the relation of this index with the Randić connectivity index as follows:

$$ \sqrt{\frac{3}{4}}\mathit{GA}(G)\geq R(G). $$

With this inequality, Theorem 12 implies the required results. □

In the coming theorem, we derive an inequality between the Randić connectivity and the third *ABC* indices.

#### Theorem 13

*Consider a connected graph* *G* *having order* $n\geq3$, *size* *m* *and diameter* $d_{G}\geq2$, *then*

$$ \mathit{ABC}_{3}(G)> \frac{m}{d_{G}}\sqrt{ \frac{d_{G}^{2}}{R(G)+1-\sqrt{2}}-2} $$

#### Proof

By considering the function $F(\varepsilon_{p},\varepsilon_{q})=\sqrt{(\varepsilon_{p}+\varepsilon _{q}-2)/\varepsilon_{p}\cdot \varepsilon_{q}}$ in (4), we have the third *ABC* index as

$$ \mathit{ABC}_{3}(G)=\sum_{pq\in E_{G}}\sqrt{ \frac{\varepsilon_{p}+\varepsilon_{q}-2}{\varepsilon _{p}\cdot \varepsilon_{q}}}. $$

From the lower bound of (9), we have the relation of this index with the diameter of *G* as follows:

$$ \frac{2m^{2}}{d_{G}^{2}}(d_{G}-1)\leq \bigl(\mathit{ABC}_{3}(G) \bigr)^{2}, $$

where $d_{G}\geq r_{G}\geq2$, and the equality holds iff *G* is a self-centered graph. It can be written as

21 $$ \frac{1}{d_{G}}-\frac{1}{d_{G}^{2}}\leq \frac{ (\mathit{ABC}_{3}(G) )^{2}}{2m^{2}}. $$

Now, from inequality (11) we have

$$ \frac{1}{2R(G)+2-2\sqrt{2}}\leq\frac{1}{d_{G}}, $$

and the equality holds iff $G\cong P_{n}$ for $n\geq3$. From this inequality, we also have

$$ \frac{1}{2R(G)+2-2\sqrt{2}}-\frac{1}{d_{G}^{2}}\leq \frac{1}{d_{G}}-\frac{1}{d_{G}^{2}}. $$

By combining it with inequality (21), we obtain

$$ \frac{1}{2R(G)+2-2\sqrt{2}}-\frac{1}{d_{G}^{2}}< \frac{ (\mathit{ABC}_{3}(G) )^{2}}{2m^{2}}, $$

where $d_{G}\geq2$ and the equality does not hold because if $G\cong P_{n}$ for $n\geq3$, then *G* cannot be a self-centered graph. After simplification, we get the required result. □

### *ABC* index in relation with distance-based indices

In the following theorem, we derive inequalities between the *ABC* index and certain distance-based indices such as eccentric connectivity, connective eccentric, augmented eccentric connectivity, and Wiener indices.

#### Theorem 14

*Consider a connected graph* *G* *having order* $n\geq2$ *and size* *m*, *then*

$$\begin{aligned} (\mathrm{a})&\quad \mathit{ABC}(G)> \frac{\xi^{c}(G)}{2m}+\frac{1}{\sqrt{2}}(n-1)- \biggl\lfloor \frac{n}{2} \biggr\rfloor , \\ (\mathrm{b})&\quad \mathit{ABC}(G)> \frac{2m}{C^{\xi}(G)}+\frac{1}{\sqrt{2}}(n-1)- \biggl\lfloor \frac{n}{2} \biggr\rfloor , \\ (\mathrm{c})&\quad \mathit{ABC}(G)> \frac{n\cdot \delta^{\delta}}{\xi^{ac}(G)}+\frac{1}{\sqrt {2}}(n-1)- \biggl\lfloor \frac{n}{2} \biggr\rfloor , \\ (\mathrm{d})&\quad 4mn\mathit{ABC}(G)> 3W(G)+2\sqrt{2}mn(n-1)-4mn \biggl\lfloor \frac{n}{2} \biggr\rfloor +3(n-1), \end{aligned}$$

*where* *δ* *represents the minimum degree of* *G*.

#### Proof

By considering the function $F(d_{p},d_{q})=\sqrt{(d_{p}+d_{q}-2)/d_{p}\cdot d_{q}}$ in the general expression (1) for degree-based indices of *G*, we have the *ABC* index of *G* as follows:

$$ \mathit{ABC}(G)=\sum_{pq\in E_{G}}\sqrt{\frac{d_{p}+d_{q}-2}{d_{p}\cdot d_{q}}}. $$

From (5) we have $d_{G}\leq2r_{G}$. Then, from (12), we obtain the relation of this index with the diameter of *G* as follows:

22 $$ d_{G}\leq 2\mathit{ABC}(G)+2 \biggl\lfloor \frac{n}{2} \biggr\rfloor -\sqrt{2}(n-1), $$

and the equality holds iff $G\cong P_{n}$ for $n\geq3$.

By combining inequality (22) with inequalities (16)–(18), we have

$$\begin{aligned}& \frac{\xi^{c}(G)}{2m}< 2\mathit{ABC}(G)+2 \biggl\lfloor \frac{n}{2} \biggr\rfloor -\sqrt{2}(n-1), \\& \frac{2m}{C^{\xi}(G)}< 2\mathit{ABC}(G)+2 \biggl\lfloor \frac{n}{2} \biggr\rfloor -\sqrt{2}(n-1), \\& \frac{n\delta^{\delta}}{\xi^{ac}(G)}< 2\mathit{ABC}(G)+2 \biggl\lfloor \frac {n}{2} \biggr\rfloor -\sqrt{2}(n-1), \end{aligned}$$

and the equality does not hold in each of these inequalities because if $G\cong P_{n}$ for $n\geq3$, then *G* cannot be a self-centered graph. After simplification, we get the required results (a), (b), and (c).

Also, from result (a) we have

$$ 2mABC(G)+2m \biggl\lfloor \frac{n}{2} \biggr\rfloor -\sqrt{2}m(n-1)> \xi^{c}(G). $$

By combining this inequality with inequality (20), we get the required result (d). □

In the coming theorem, we derive an inequality between the *ABC* and the third *ABC* indices.

#### Theorem 15

*Consider a connected graph* *G* *having order* $n\geq2$, *size* *m*, *and diameter* $d_{G}\geq2$, *then*

$$ \mathit{ABC}_{3}(G)>\frac{m}{d_{G}}\sqrt{ \frac{d_{G}^{2}}{\mathit{ABC}(G)+ \lfloor \frac{n}{2} \rfloor-\frac{1}{\sqrt{2}}(n-1)}-2}. $$

#### Proof

From (22), we have

$$ \frac{1}{2\mathit{ABC}(G)+2 \lfloor\frac{n}{2} \rfloor-\sqrt {2}(n-1)}\leq \frac{1}{d_{G}}, $$

and the equality holds iff $G\cong P_{n}$ for $n\geq3$.

From this inequality, we also have

$$ \frac{1}{2\mathit{ABC}(G)+2 \lfloor\frac{n}{2} \rfloor-\sqrt {2}(n-1)}-\frac{1}{d_{G}^{2}}\leq \frac{1}{d_{G}}-\frac{1}{d_{G}^{2}}. $$

By combining it with inequality (21), we obtain

$$ \frac{1}{2\mathit{ABC}(G)+2 \lfloor\frac{n}{2} \rfloor-\sqrt {2}(n-1)}-\frac{1}{d_{G}^{2}}< \frac{ (\mathit{ABC}_{3}(G) )^{2}}{2m^{2}}, $$

where $d_{G}\geq2$ and the equality does not hold because if $G\cong P_{n}$ for $n\geq3$, then *G* cannot be a self-centered graph. After simplification, we get the required result. □

### Harmonic index in relation with distance-based indices

In the following theorem, we derive the inequalities between the harmonic index and certain distance-based indices such as eccentric connectivity, connective eccentric, augmented eccentric connectivity, and Wiener indices for any non-self-centered graph.

#### Theorem 16

*Consider a non*-*self*-*centered graph* *G* *having order* *n* *and size* *m*, *then*

$$\begin{aligned} (\mathrm{a})&\quad H(G)< \frac{\xi^{c}(G)}{m}+\frac{n}{2}-1, \\ (\mathrm{b})&\quad H(G)< \frac{4m}{C^{\xi}(G)}+\frac{n}{2}-1, \\ (\mathrm{c})&\quad H(G)< \frac{2n\cdot \Delta^{\Delta}}{\xi^{ac}(G)}+\frac{n}{2}-1, \\ (\mathrm{d})&\quad mn(n-1)H(G)> \frac{\sqrt{3}}{4}W(G)+\frac{2}{\sqrt{3}}m( \sqrt{2}-1)+\frac{\sqrt{3}}{4}(n-1), \end{aligned}$$

*where* Δ *represents the maximum degree*.

#### Proof

For a non-self-centered graph *G*, from (5) we have $r_{G}< d_{G}\leq2\cdot r_{G}$. Then from the compound inequalities (6)–(8), we obtain the relation of the diameter of *G* with the eccentric connectivity, connective eccentric, and augmented eccentric connectivity indices as follows:

23 $$\begin{aligned}& d_{G}\leq\frac{\xi^{c}(G)}{m}, \end{aligned}$$

24 $$\begin{aligned}& d_{G}\leq\frac{4m}{C^{\xi}(G)}, \end{aligned}$$

25 $$\begin{aligned}& d_{G}\leq\frac{2n\cdot \Delta^{\Delta}}{\xi^{ac}(G)}. \end{aligned}$$

Now, by considering the function $F(d_{p},d_{q})=2/(d_{p}+d_{q})$ in the general expression of degree-based indices as given in (1), we have the harmonic index of *G* as follows:

$$ H(G)=\sum_{pq\in E_{G}}\frac{2}{d_{p}+d_{q}}. $$

From (13), we obtain the relation of this index with the diameter of *G* as follows:

26 $$ H(G)-\frac{n}{2}+1\leq d_{G}, $$

and the equality holds iff $G\cong K_{n}$ for $n\geq4$.

By combining inequality (26) with inequalities (23)–(25), we obtain

$$\begin{aligned}& H(G)-\frac{n}{2}+1< \frac{\xi^{c}(G)}{m}, \\& H(G)-\frac{n}{2}+1< \frac{4m}{C^{\xi}(G)}, \\& H(G)-\frac{n}{2}+1< \frac{2n\cdot \Delta^{\Delta}}{\xi^{ac}(G)}. \end{aligned}$$

The equality does not exist in each of these inequalities because the complete graph $K_{n}$ is a self-centered graph. After simplification, we get the required results (a), (b), and (c).

Also, from the compound inequality (15), we obtain

27 $$ (n-1)H(G)\geq \mathit{GA}(G), $$

and from the result (d) of Corollary 1, we have

$$ \mathit{GA}(G)>\frac{\sqrt{3}}{4m}W(G)+\frac{2}{\sqrt{3}}(\sqrt{2}-1)+ \frac{\sqrt {3}}{4m}(n-1). $$

By combining this inequality with inequality (27), we get the required result (d). □

In the coming theorem, we present the inequality relations of the harmonic index with eccentric connectivity and connective eccentric indices for self-centered graphs.

#### Theorem 17

*Consider a self*-*centered graph* *G* *having order* $n\geq4$ *and size* *m*, *then*

$$\begin{aligned} (\mathrm{a})&\quad H(G)\leq\frac{\xi^{c}(G)}{2m}+\frac{n}{2}-1, \\ (\mathrm{b})&\quad H(G)\leq\frac{2m}{C^{\xi}(G)}+\frac{n}{2}-1, \end{aligned}$$

*and the equality holds iff* $G\cong K_{n}$.

#### Proof

For a self-centered graph *G*, from (5) we have $d_{G}=r_{G}$. Then, from the compound inequalities (6)–(7), we obtain the equality relation of the diameter of *G* with the eccentric connectivity, connective eccentric, and augmented eccentric connectivity indices as follows:

$$\begin{aligned}& d_{G}=\frac{\xi^{c}(G)}{2m}, \\& d_{G}=\frac{2m}{C^{\xi}(G)}. \end{aligned}$$

By using these relations in inequality (26), we get the required results. □

In the following theorem, we establish an inequality between harmonic and augmented eccentric connectivity indices for regular self-centered graphs.

#### Theorem 18

*Consider a regular self*-*centered graph* *G* *of degree* *d* *having order* $n\geq4$ *and size* *m*, *then*

$$ H(G)\leq\frac{nd^{d}}{\xi^{ac}(G)}+\frac{n}{2}-1, $$

*and the equality holds iff* $G\cong K_{n}$.

#### Proof

For a self-centered graph *G*, from (5) we have $d_{G}=r_{G}$. Then, from the compound inequality (8), we obtain the equality relation of the diameter of *G* with the augmented eccentric connectivity indices as follows:

$$ d_{G}=\frac{nd^{d}}{\xi^{ac}(G)}. $$

By using this relation in inequality (26), we get the required result. □

In the coming theorem, we derive an inequality relation between harmonic and third *ABC* indices for non-self-centered graphs.

#### Theorem 19

*Consider a non*-*self*-*centered graph* *G* *having order* $n\geq4$, *size* *m*, *and radius* $r_{G}\geq2$, *then*

$$ \mathit{ABC}_{3}(G)< \frac{m}{r_{G}}\sqrt{ \frac{8r_{G}^{2}}{2H(G)-n+2}-2}. $$

#### Proof

For a non-self-centered graph *G*, from (5) we have $r_{G}< d_{G}\leq2r_{G}$. By using this relation, we obtain the inequality

28 $$ \frac{1}{r_{G}}-\frac{1}{r_{G}^{2}}\leq \frac{2}{d_{G}}- \frac{1}{r_{G}^{2}}. $$

Now, from the compound inequality (9), we have

$$ \bigl(\mathit{ABC}_{3}(G) \bigr)^{2}\leq \frac{2m^{2}}{r_{G}^{2}} (r_{G}-1 ). $$

It can be written as

29 $$ \frac{1}{2m^{2}} \bigl(\mathit{ABC}_{3}(G) \bigr)^{2}\leq \frac{1}{r_{G}}-\frac{1}{r_{G}^{2}}, $$

where $r_{G}\geq2$, and the equality holds iff *G* is a self-centered graph.

By combining inequality (28) with inequality (29), we have

30 $$ \frac{1}{4m^{2}} \bigl(\mathit{ABC}_{3}(G) \bigr)^{2}+\frac{1}{2r_{G}^{2}}< \frac {1}{d_{G}}, $$

and the equality does not hold because *G* is a non-self-centered graph.

Also, from (13) we have

31 $$ \frac{1}{d_{G}}\leq\frac{1}{H(G)-\frac{n}{2}+1}, $$

and the equality holds if and only if $G\cong K_{n}$ for $n\geq4$.

By combining inequality (31) with inequality (30), we obtain

$$ \frac{1}{4m^{2}} \bigl(\mathit{ABC}_{3}(G) \bigr)^{2}+ \frac{1}{2r_{G}^{2}}< \frac {1}{H(G)-\frac{n}{2}+1}. $$

After simplification, we get the required result. □

In the following theorem, we derive an inequality between harmonic and third *ABC* indices for self-centered graphs.

#### Theorem 20

*Consider a self*-*centered graph* *G* *having order* $n\geq4$, *size* *m*, *and diameter* $d_{G}\geq2$, *then*

$$ \mathit{ABC}_{3}(G)< \frac{m}{d_{G}}\sqrt{ \frac{4d_{G}^{2}}{2H(G)-n+2}-1}. $$

#### Proof

For a self-centered graph *G*, we have $d_{G}=r_{G}$. Then, from the compound inequality (9), we have

32 $$ \frac{1}{2m^{2}} \bigl(\mathit{ABC}_{3}(G) \bigr)^{2}=\frac{1}{d_{G}}-\frac{1}{d_{G}^{2}}, $$

where $d_{G}\geq2$.

Also, from inequality (31) we have

$$ \frac{1}{d_{G}}-\frac{1}{d_{G}^{2}}\leq\frac{1}{H(G)-\frac {n}{2}+1}-\frac{1}{d_{G}^{2}}, $$

and the equality holds iff $G\cong K_{n}$ for $n\geq4$.

By using (32), we obtain

$$ \frac{1}{2m^{2}} \bigl(\mathit{ABC}_{3}(G) \bigr)^{2}< \frac{1}{H(G)-\frac {n}{2}+1}-\frac{1}{d_{G}^{2}}, $$

where $d_{G}\geq2$, and the equality does not hold because if $G\cong K_{n}$ for $n\geq4$, then $d_{G}=1$. After simplification, we get the desired result. □

## Conclusion

In this paper, some inequality relations have been studied between two topological indices belonging to degree-based and distance-based indices. We derived the relations of Randić connectivity, *GA*, *ABC*, and harmonic indices with eccentric connectivity, connective eccentric, augmented eccentric connectivity, Wiener, and third *ABC* indices. Our derived inequality relations can be very helpful in the relative study of these indices.
